# Supplementary material for: Recombinant Escherichia coli BL21 with LngA Variants from ETEC E9034A Promotes Adherence to HT-29 Cells
Source: Pathogens. 2023 Feb 16;12(2):337. doi: 10.3390/pathogens12020337 (PMC9962868; doi:10.3390/pathogens12020337)
Supplement: Supplementary file 1 [file pathogens-12-00337-s001.zip › pathogens-2106837-supplementary.pdf]

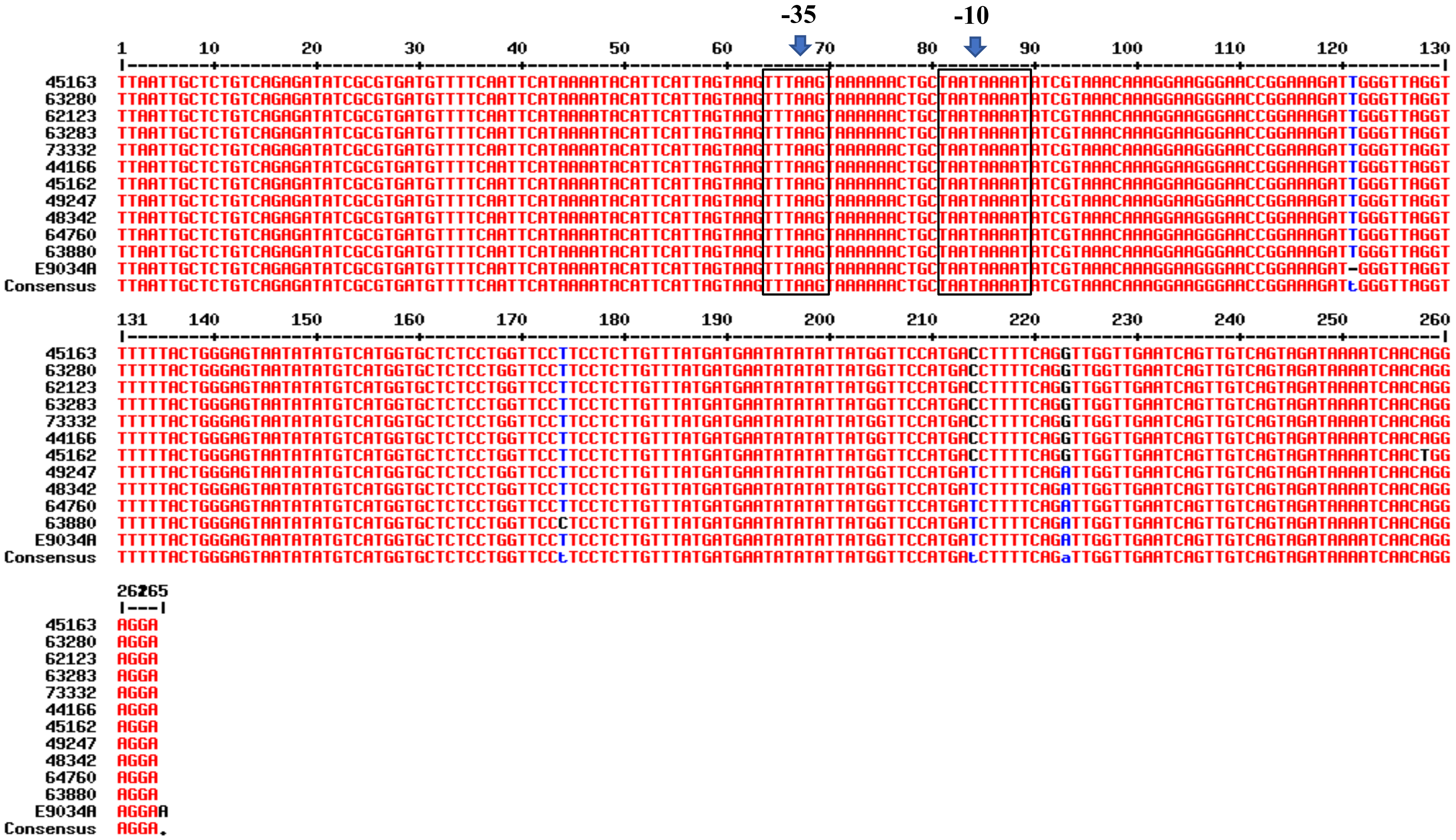

Figure S1. Alignment of the *lngA* nucleotide sequence promoter region. The RNA polymerase-binding sites were determined to be at positions -10 and -35.

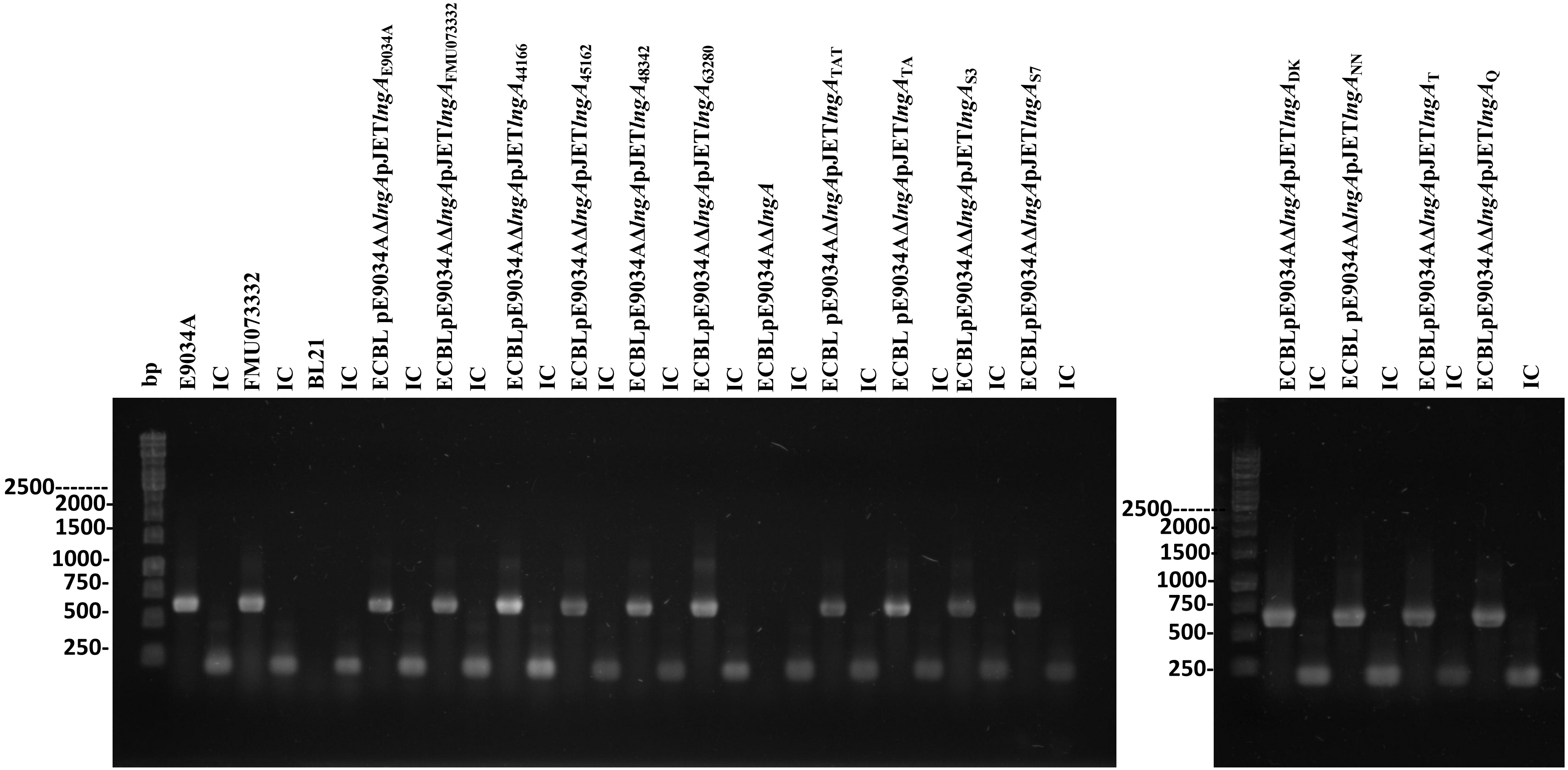

Figure S2. RT–PCR assays for *lngA* gene detection. Clinical ETEC strains (E9034A and FMU73332), ECBL (*E. coli* BL21) and recombinant ECBL strains with *lngA* variants and site-specific mutations. IC: internal control, the ARN 16S gene.

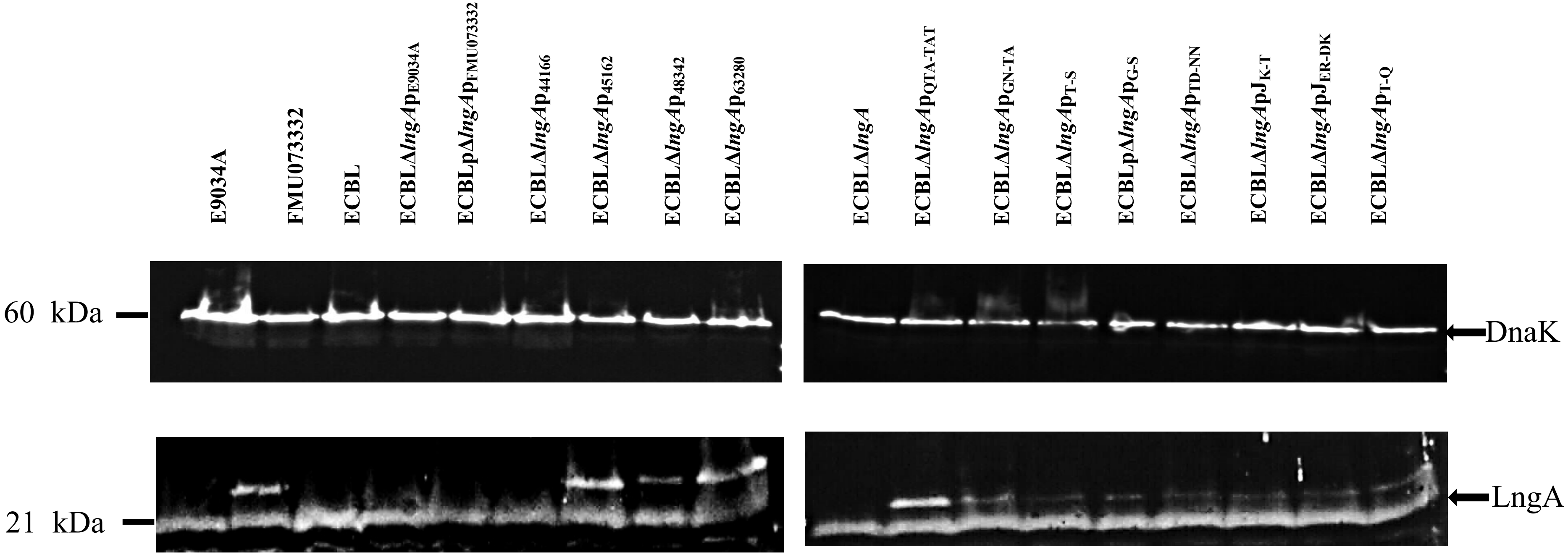

Figure S3. Immunodetection by Western blotting of clinical ETEC strains (E9034A and FMU073332), ECBL (*E. coli* BL21), recombinant ECBL strains with *lngA* variants and sitespecific mutation in *lngA* strains.

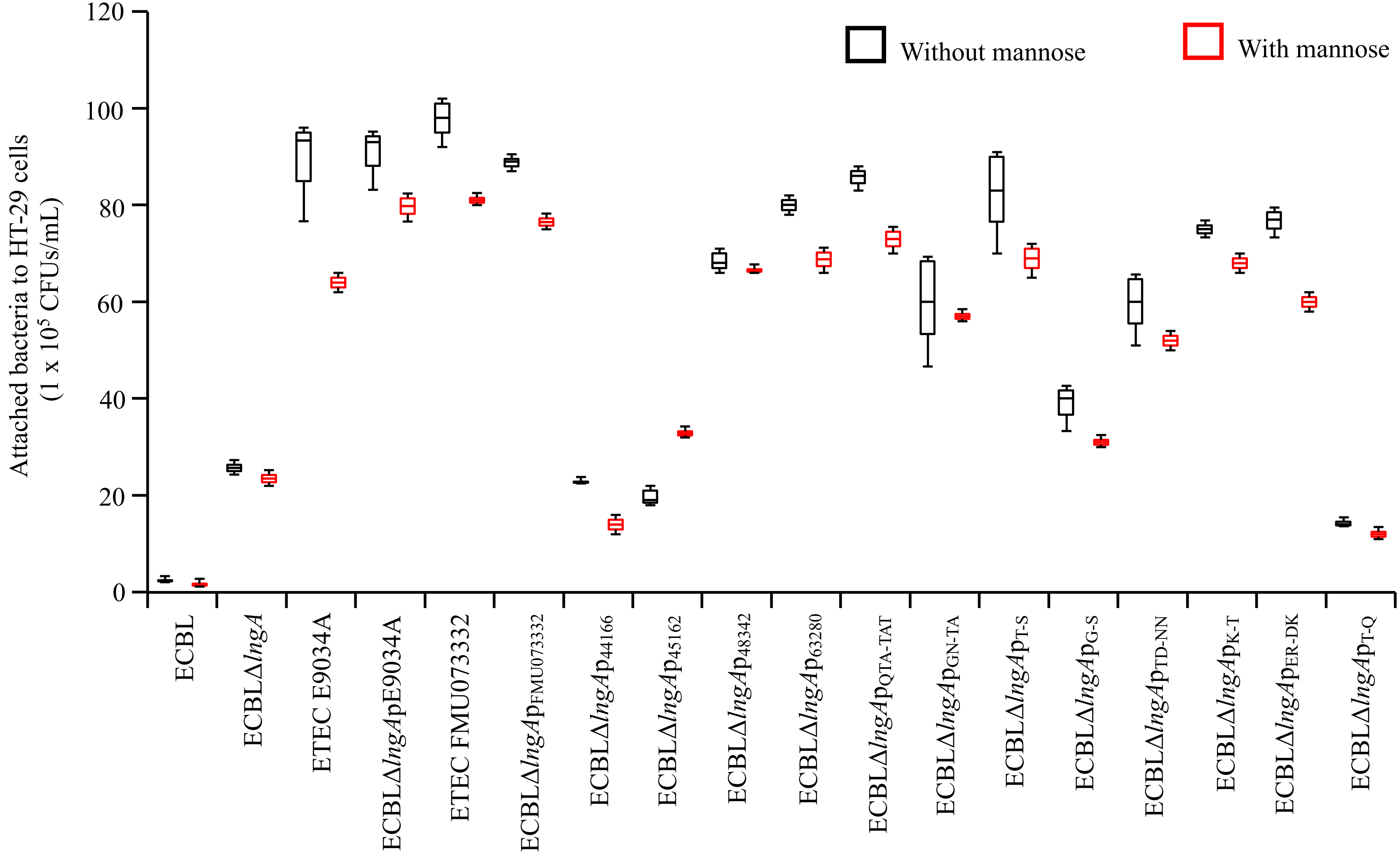

Figure S4. Quantitative analysis of bacterial adherence to HT-29 cells. ETEC (E9034A and FMU07332), ECBL (*E. coli* BL21), recombinant ECBL strains with *lngA* variants and site-specific mutations. Adherence assays were performed with and without mannose with 6 h of incubation at 37°C. In addition, 1% mannose was added to 1 mL (final volume) of DMEM per well before infection with each strain.

ECBLΔ*lngA*

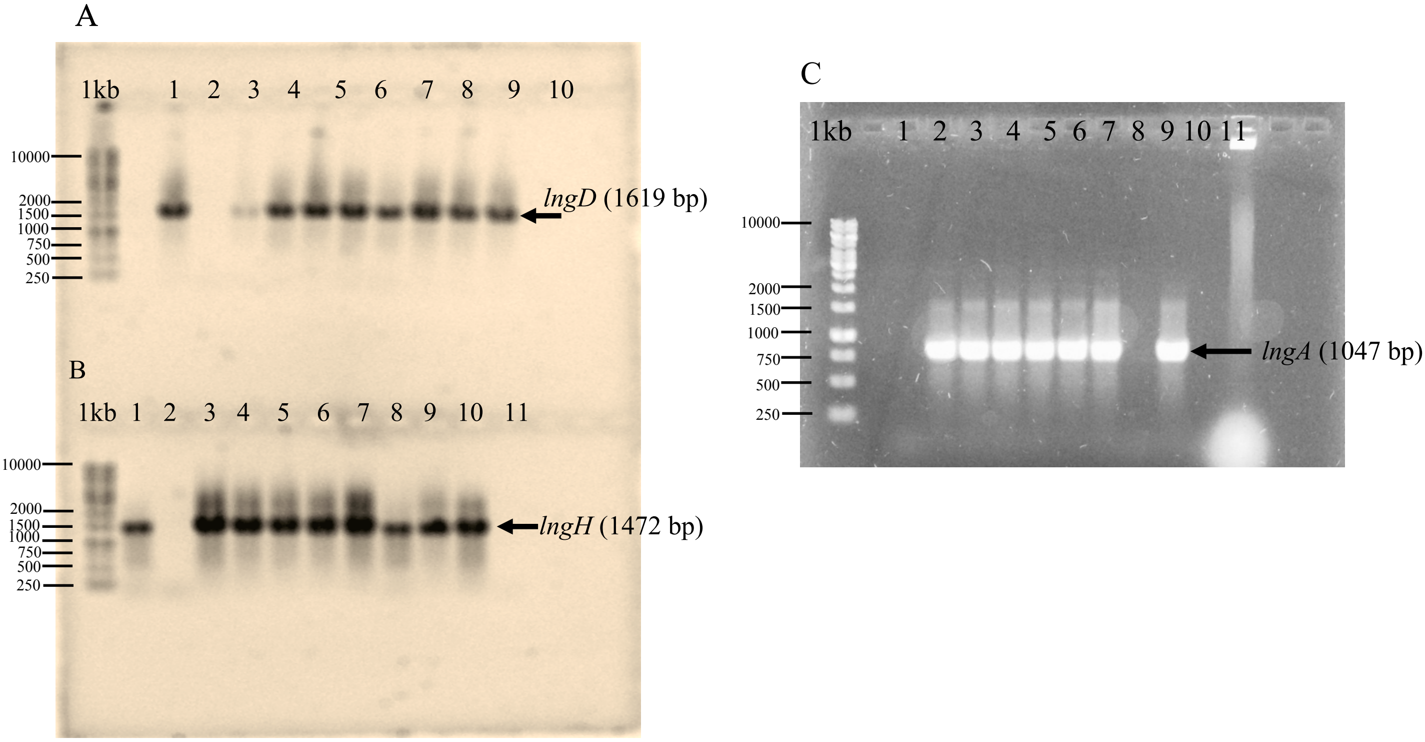

Figure S5. PCR asayas for the *lngD*, *lngH* and *lngA*. (A) Agarose gel stained with ethidium bromide revealing size differences in the PCR amplification products of the *lng D* gene (1619 bp). Line 1: E9034A. Lines 2 to 9: ECBLΔ*lngA* bacterial colonies to confirm *lng* operon insertion. Line 10: Negative control. (B) Agarose gel stained with ethidium bromide revealing size differences in the PCR amplification products of the *lng H* gene (1472 bp). Line 1: E9034A. Lines 2 to 10: ECBLΔ*lngA* bacteria colonies to confirm *lng* operon insertion. Line 11: Negative control. (C) Agarose gel stained with ethidium bromide revealing size differences in the PCR amplification product *lngA* gene (1047 bp). Line 1: ECBLΔ*lngA*. Line 2: E9034A. Lines 3 to 10: ECBLΔ*lngA* bacteria colonies to confirm lng operon insertion. Line 11: Negative control.
